# Raman study on zinc-blende single InAs nanowire grown on Si (111) substrate

**DOI:** 10.1186/1556-276X-8-27

**Published:** 2013-01-14

**Authors:** Tianfeng Li, Lizhen Gao, Wen Lei, Lijun Guo, Tao Yang, Yonghai Chen, Zhanguo Wang

**Affiliations:** 1 Department of Physics, School of Physics and Electronics, Henan University, Kaifeng, 475004, People’s Republic of China; 2 Key Laboratory of Semiconductor Material Science, Institute of Semiconductors, Chinese Academy of Sciences, Beijing, 100083, People’s Republic of China; 3 School of Electrical, Electronic and Computer Engineering, The University of Western Australia, 35 Stirling Hwy, Crawley, 6009, Australia

**Keywords:** Nanowires (NWs), Raman spectroscopy, Phonon property, Polarize, 62.23.Hj, 81.07.Gf, 63.22.Gh, 61.46.Km

## Abstract

We report polarized Raman scattering studies on single InAs nanowires (NWs). The NWs were grown by metalorganic chemical vapor deposition on Si (111) substrates without external catalyst and showed a zinc-blende crystal structure. The single NWs were studied for different polarization excitation of the incident laser beam relative to the NW axis. The transverse optical (TO) mode exhibits maximum intensity when both the incident and analyzed light polarizations are parallel to the NW axis. The TO mode of InAs NWs is found to act like a nearly perfect dipole antenna, which can be attributed to the one-dimensional NW geometry and Raman selection rules.

## Background

Semiconductor nanowires (NWs) have been intensively studied in the last decade due to their novel physical properties and potential applications in high-performance devices, such as field-effect transistors, lasers, photodetectors, and photovoltaic devices [1-5]. Among them, InAs NWs possess excellent electron transport properties such as high bulk mobility, small effective mass, and low ohmic contact resistivity, which can be used for making high-performance electronic devices such as high-mobility transistors [6-8]. For their device applications, it is important to understand the physical properties of these InAs NWs, including phonon scattering information. Although NWs with low defect density have been reported, many NW material systems suffer from various types of planar defects, predominantly rotational twins and twinning superlattices, alternating zinc-blende (ZB)/wurtzite polytypes, as well as point defects [9-12]. Raman scattering, a nondestructive contactless characterization technique, provides an effective approach to probe phonon properties. Combined with advanced confocal microscopy, Raman scattering can be well used to investigate the phonon properties of single NWs with a spatial resolution of roughly half the excitation wavelength. Phonon energies, scattering cross sections, and symmetry properties of optical phonons are determined by analyzing inelastically scattered light, providing information about crystal structure and composition, electronic properties, and electron–phonon and phonon-phonon interactions [13]. In the meantime, Raman scattering in NWs is expected to be different from that in their bulk materials due to their one-dimensional geometry [14], where the polarized excitation will show a significant effect on phonon modes. Indeed, some previous studies on NWs do show an obvious polarization effect [15-20]. Though some works [21,22] have reported on the Raman spectra of InAs NW assemblies, little attention has been devoted to the Raman scattering in single InAs NWs [23,24], especially the effect of excitation polarization on phonon vibration. In this work, we present a Raman study on single zinc-blende InAs NWs. The effect of excitation polarization on the phonon properties of single InAs NWs is also investigated in detail.

## Methods

### Experimental details

**Figure 1:**
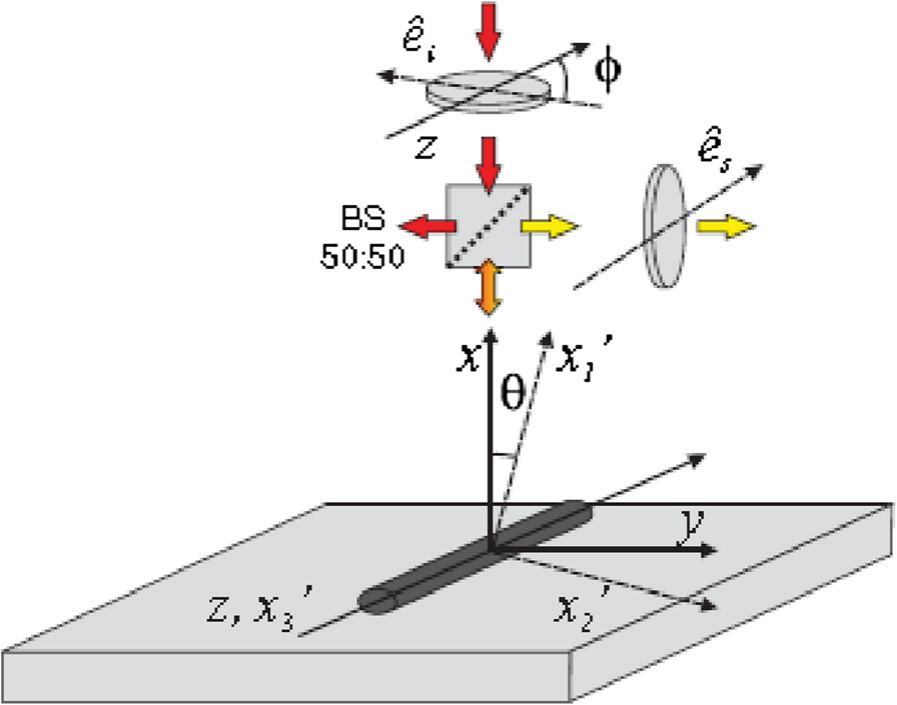
**Sketch of the experimental setup and the used coordinate systems (*x,y,z*) and (*x***^**′**^_**1**_**, *x***^**′**^_**2**_**, *x***^**′**^_**3**_**) in backscattering geometry.**${\hat{e}}_{i}$ and ${\hat{e}}_{s}$ are the incident and scattered light polarizations, respectively.

The InAs NWs were grown catalyst-free by metalorganic chemical vapor deposition (Thomas Swan Scientific Equipment, Ltd., Cambridge, UK) on Si (111) substrates. The InAs NWs investigated here were from a characteristic sample grown for 7 min under a growth temperature of 550°C and a V/III ratio of 100 (the growth details were reported elsewhere) [21]. The NWs are crystalline having high-density twins and stacking faults over the entire nanowire length, 40 to 60 nm in diameter, and up to 5 μm in length. The epitaxial relationship between the InAs NWs and Si (111) substrate and the predominant crystal structure of these NWs were analyzed by X-ray diffraction (XRD) and transmission electron microscopy (TEM; Tecnai F20, 200 KeV, FEI, Eindhoven, The Netherlands). Raman scattering in InAs NWs was performed in backscattering geometry at room temperature with a Jobin–Yvon HR800 (Horiba Ltd., Longjumeau, France) confocal micro-Raman system. To measure the Raman scattering in single NWs, InAs NWs were removed from the sample surface and transferred to a graphite crystal (highly ordered pyrolytic graphite (HOPG)). The single InAs NWs were excited using the 514.5-nm Ar^+^ laser line to a 1-μm spot on the surface with an excitation power of 2.5 mW. The excitation polarization-dependent Raman scattering in single NWs was performed using the method shown in [23], and the schematic diagram of the setup is shown in Figure 1. First, the incoming laser beam passes through a *λ*/2 plate so that its polarization ${\hat{e}}_{i}$ can be rotated by an angle *ϕ*. After passing through a beam splitter (50:50), it is focused on the nanowire with an objective of ×100 (NA 0.9). The polarization state of the scattered light ${\hat{e}}_{s}$ is analyzed by measuring the intensity of the two components (parallel or perpendicular to the wire). For this, a polarizer is used. Two coordinate systems are introduced: the laboratory coordinate system (*x*, *y*, *z*) and the crystal coordinate system of the NW (*x*′_1_, *x*′_2_, *x*′_3_). *z* and *x*′_3_ are parallel to the growth axis of the NW, while *x*′_1_ (*x*′_2_) is rotated by an angle (*θ*) with respect to the *x*(*y*) axis in the *x* - *y* plane.

### Theoretical considerations of zinc-blende InAs

In the Raman scattering experiment, the scattering intensities *I*_s_ can be calculated from the Raman tensor which depends on the crystal symmetry as [23,25,26]:

(1) $$I_{\text{s}}\propto {\left|{\hat{e}}_{i}\cdot R\cdot {\hat{e}}_{s}\right|}^{2}\text{,}$$

where *R* is the Raman tensor and ${\hat{e}}_{i}$ and ${\hat{e}}_{s}$ are the polarization of the incident radiation and the scattered radiation, respectively. The zone-center optical phonon in the zinc-blende structure is split into a doubly degenerate transverse optical (TO) mode and a longitudinal optical (LO) mode, and the Raman tensor elements are different for the TO and LO modes. As calculated, the TO mode can be observed in backscattering from the (110) and (111) surfaces, while the LO mode is allowed from the (100) and (111) surfaces [16].

In this work, we investigated single InAs NWs grown in the [111] (zinc-blende) direction. We set

(2) $$\begin{array}{l} x_{1}^{'}=T^{-1}{\hat{e}}_{1}=\frac{1}{\sqrt{2}}\left[1,\bar{1},0\right],x_{2}^{'}=T^{-1}{\hat{e}}_{2}\\ \;=\frac{1}{\sqrt{6}}\left[1,1,\bar{2}\right],\mathrm{and}\;x_{3}^{'}=T^{-1}{\hat{e}}_{3}=\frac{1}{\sqrt{3}}\left[1,1,1\right]\text{,} \end{array}$$

representing the basis of the NW crystal coordinate system. When an optical phonon is polarized along the direction ${\hat{e}}_{1}=\left(100\right)∥x$, ${\hat{e}}_{2}=\left(010\right)∥y$, or ${\hat{e}}_{3}=\left(001\right)∥z$, its Raman tensors $R_{e_{1}}$, $R_{e_{2}}$, and $R_{e_{3}}$ will have only two nonzero components (*d*), which can be represented by a (3 × 3) matrix:

(3) $$\begin{array}{l} R_{e_{1}}=\left(\begin{array}{ccc} 0 & 0 & 0\\ 0 & 0 & d\\ 0 & d & 0 \end{array}\right),R_{e_{2}}=\left(\begin{array}{ccc} 0 & 0 & d\\ 0 & 0 & 0\\ d & 0 & 0 \end{array}\right),\\ R_{e_{3}}=\left(\begin{array}{ccc} 0 & d & 0\\ d & 0 & 0\\ 0 & 0 & 0 \end{array}\right)\text{,} \end{array}$$

respectively [23].

In order to calculate the selection rules for the zinc-blende structure, the Raman tensors are transformed in two steps. First, the Raman tensors are transformed into the laboratory coordinate system with the basis $\left\{{\hat{e}}_{1},{\hat{e}}_{2},{\hat{e}}_{3}\right\}$. Secondly, they are rotated around the *z* axis by the angle *θ* (see Figure 1) in order to account for the additional degree of freedom of the top surface of the NWs. The two transformations can be described by the matrices

(4) $$\begin{array}{l} T=\left(\begin{array}{ccc} \frac{1}{\sqrt{2}} & -\frac{1}{\sqrt{2}} & 0\\ \frac{1}{\sqrt{6}} & \frac{1}{\sqrt{6}} & \frac{-2}{\sqrt{6}}\\ \frac{1}{\sqrt{3}} & \frac{1}{\sqrt{3}} & \frac{1}{\sqrt{3}} \end{array}\right),\\ S=\left(\begin{array}{ccc} \cos\theta & -\sin\theta & 0\\ \sin\theta & \cos\theta & 0\\ 0 & 0 & 1 \end{array}\right)\text{,} \end{array}$$

where *T* denotes the transformation into the basis $\left\{{\hat{e}}_{1},{\hat{e}}_{2},{\hat{e}}_{3}\right\}$ and *S* is the rotation about the NW *z* axis. For reasons of simplicity, we define *M* = *ST*. The Raman tensors $R_{{x^{\prime }}_{i}}$ for displacements along the directions *x*′_*i*_ in the basis $\left\{{\hat{e}}_{1},{\hat{e}}_{2},{\hat{e}}_{3}\right\}$ can now be written as

(5) Rxi'=∑j=13MijRej,i=1,2,3,

and the Raman tensors ${\tilde{R}}_{{x^{\prime }}_{i}}$ in the basis $\left\{{{\hat{x}}^{\prime }}_{1},{{\hat{x}}^{\prime }}_{2},{{\hat{x}}^{\prime }}_{3}\right\}$ can be described by

(6) $${\tilde{R}}_{{x^{\prime }}_{i}}=MR_{{x^{\prime }}_{i}}M^{T},i=1,2,3.$$

**Figure 2:**
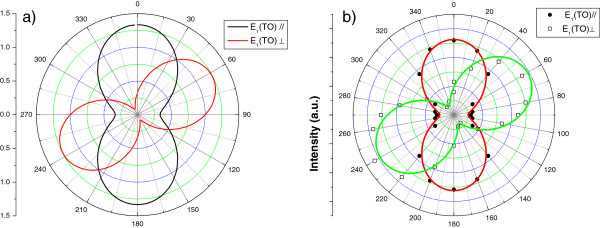
**Calculated intensity polar patterns of scattered light and measured polarized Raman scattering of TO phonon.** (**a**) Calculated intensity polar patterns of the scattered light polarized perpendicular (*I*_⊥_) or parallel (*I*_∥_) to the [111] direction as a function of the angle *ϕ* of the incident polarization with respect to [111] is shown for TO phonons in backscattering from a bulk InAs (110) substrate. (**b**) Measured polarized Raman scattering of the TO mode on a reference bulk InAs (110) substrate. Spheres and open squares represent the parallel and perpendicular components of the Raman signal, respectively. The continuous line is a squared sine fit to the data.

Here, we have considered a backscattering configuration along the *x* axis. In laboratory coordinates, the polarization ${\hat{e}}_{i}$ of the incident radiation and the polarization ${\hat{e}}_{s}$ of the scattered light take the form (see Figure 1)

(7) $${\hat{e}}_{\text{i}}=\left(\begin{array}{c} 0\\ \sin\phi \\ \cos\phi \end{array}\right),{\hat{e}}_{\text{s}}^{⊥}=\left(\begin{array}{c} 0\\ 1\\ 0 \end{array}\right),{\hat{e}}_{\text{s}}^{∥}=\left(\begin{array}{c} 0\\ 0\\ 1 \end{array}\right)\text{,}$$

depending on whether the scattered radiation is analyzed perpendicular (${\hat{e}}_{s}^{⊥}$) or parallel (${\hat{e}}_{s}^{∥}$) to the wire axis, respectively. By inserting the obtained Raman tensors (Equation 5) in Equation 1, the Raman intensities of the zinc-blende structure for different configurations can be obtained. As shown in Figure 2, the theoretical intensities of the scattered light polarized perpendicular (*I*_⊥_, polarized in the *y* direction) or parallel (*I*_∥_, polarized in the *z* direction) to the [111] direction as a function of the angle *ϕ* of the incident polarization with respect to [111] are shown for TO (Figure 2a) from a bulk InAs substrate (110) in polar plots taking into account only the contribution of the Raman tensors. For perpendicular analysis (*I*_⊥_), the maximum intensity of the TO mode is obtained for an angle of the incident polarization of 63°, while for the parallel analyzed polarization (*I*_∥_), the maximum intensity is found for 0° with respect to the [111] direction. For reference, polarized Raman scattering was performed on a bulk InAs (110) substrate. The polar scan of the Raman intensity of the TO phonon is shown in Figure 2b. The experimental data show good agreement with the theory. The small shift of the TO intensity maxima of about 2° is attributed to an inclination of the polarization direction of the light with respect to the crystallographic axes of the substrate. It should be pointed out here that LO scattering is forbidden in this scattering configuration.

In order to calculate the polar patterns of *I*_s_ for NWs, one has to take into account the additional degree of freedom associated with the rotation of *θ* around the NW axis since it can influence the polar patterns of the optical modes. Based on [23], this angular dependence is a clear signature of the presence of zinc-blende TO modes and can be used for their assignation.

## Results and discussion

**Figure 3:**
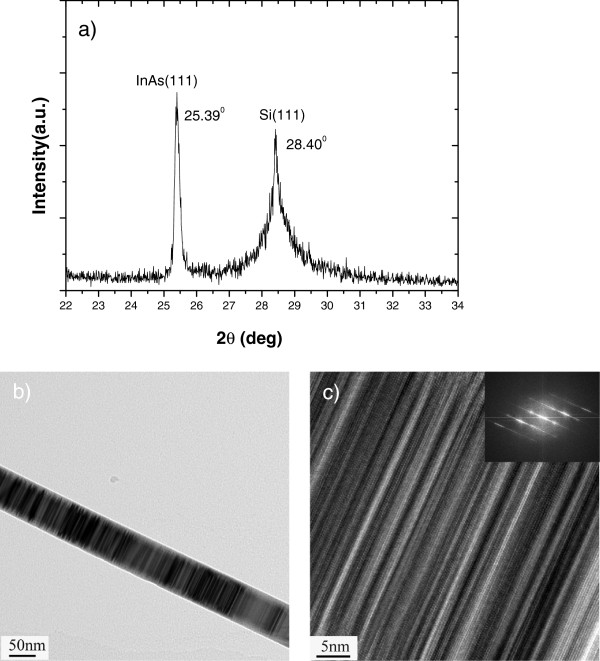
**XRD scan, low-resolution TEM, and HRTEM of a selected InAs nanowire array sample.** (**a**) XRD scan of a selected InAs nanowire array sample, confirming the epitaxial relationship between InAs (111) and Si (111) substrate. (**b**) Low-resolution TEM image of the nanowire. (**c**) HRTEM image of a portion of the nanowire. The inset of (c) shows the fast Fourier transform of the selected area, which is viewed along the [0–11] direction.

The epitaxial relationship between the InAs NWs and Si (111) substrate and the predominant crystal structure of these NWs were analyzed by XRD and TEM (Figure 3). The out-of-plane symmetric XRD 2*θ* − *ω* scan shown in Figure 3a, which was obtained from the as-grown NWs, indicates that NWs were grown epitaxially on the Si substrate. Besides the <111> reflection of Si at 28.4°, another reflection at 25.4° represented (111) of InAs. The weak peak of Si (111) may be due to not compensating for the 3.28° miscut of the Si substrate. Representative high-resolution TEM (HRTEM) images of these nanowires are presented in Figure 3b,c. Stripes with different contrast are observed along the nanowires. Careful analysis indicates that these correspond to the twin defects perpendicular to the growth axis. The detail of such defect is presented in Figure 3b. Figure 3c shows the HRTEM image of a NW with its inset showing the fast Fourier transform (FFT) image. The HRTEM image combined with the FFT image indicates that the InAs NW has a cubic, zinc-blende structure and grows along the <111> direction normal to the Si (111) substrate. The growth axis remains parallel to the (111) *B* direction.

**Figure 4:**
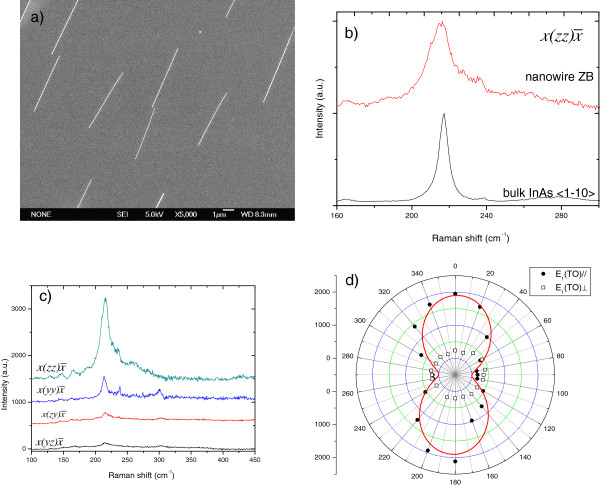
**SEM image of InAs NWs, polarized Raman spectra, and azimuthal dependence of the TO mode.** (**a**) SEM image of InAs NWs transferred on a Si substrate. (**b**) Parallel polarized Raman spectra from a bulk InAs (110) and an InAs nanowire. For both measurements, the exciting and scattered light are polarized along the <111> direction. (**c**) A series of parallel and perpendicularly polarized Raman spectra obtained using exciting light polarized parallel and perpendicular to the nanowire axis. The spectra have been shifted vertically. (**d**) Azimuthal dependence of the TO mode related to the ZB structure in the nanowire. Spheres and open squares represent the parallel and perpendicular components of the Raman signal collected with respect to the nanowire axis, respectively. The continuous line is a squared sine fit to the data.

Prior to the Raman investigations on single InAs NWs, scanning electron microscopy (SEM) measurements were performed in order to determine the shape, diameter, and length of the NWs after transfer (Figure 4a). The SEM image of InAs NWs transferred to the HOPG substrate shows that the NWs are monodisperse and well separated from each other. The NWs are 40 to 60 nm in diameter and up to 5 μm in length.

Raman measurements were performed in a backscattering configuration on single InAs NWs and from the (110) surface of a bulk InAs single crystal as reference. The general measurement geometry for a single NW is shown in Figure 1. The laboratory coordinate system *x*, *y*, *z* is chosen according to the NW geometry and the basis of the NW crystal coordinate system: ($\hat{x}\|\left[1\bar{1}0\right],\hat{y}\|\left[11\bar{2}\right],\hat{z}\|\left[111\right]$). Based on the calculated selection rules in [16], the TO phonon mode can be observed in the backscattering from the (110) and (111) InAs surfaces, while the LO phonon mode can be observed from the (100) and (111) InAs surfaces. The Raman spectra of the single InAs NW and bulk InAs obtained are shown in Figure 4b, which are measured under the configuration $x\left(z,z\right)\bar{x}$. The coordinates *y* and *z* are chosen perpendicular and parallel to the NW growth axis, respectively. Incident and scattered light polarizations were selected parallel to the NW growth axis. The Raman spectra of both nanowire and bulk InAs have been normalized with respect to the intensity of the TO phonon mode of bulk InAs for easy comparison. For bulk InAs (110), the TO mode is found at 217.2 cm^−1^[24]. The Raman scattering spectrum of InAs NWs is composed mainly by the TO mode at 215.8 cm^−1^, slightly lower than that for the reference bulk InAs (110) sample. In addition, the LO mode of the single NW is also visible at around 236 cm^−1^, the appearance of which might be caused by the disorder and an imperfect scattering geometry [24]. In addition, the TO mode of InAs NWs exhibits a downshift of about 2 to 3 cm^−1^ compared to the TO mode of bulk ZB InAs. Along with the downshift, a remarkable increase of the full width at half maximum to 14 cm^−1^ is observed. It should be mentioned that the downshift of the TO mode was also observed in the Raman measurements on the as-grown NW ensemble samples. Generally, there are two factors which might induce the downward shift of phonon mode frequency and the broadening of the Raman peak. One is laser heating effect. As reported before [27-30], local heating might also cause the downshift of phonon mode frequency and the broadening of phonon peak. To reduce the laser heating effect, we use the lowest laser power and the monodisperse wires were placed on high thermal conductivity HOPG to avoid substrate effects. An excitation power-dependent Raman measurement was performed on the single NWs, and no shifting of the phonon peak was observed when the excitation power is 0.05 mW (data not shown here), which may be due to high-thermal conductivity substrate (HOPG) and low nanowire coverage over the substrate [31]. Thus, this heating effect can be lowered in our measurements; the other is quantum confinement effect. It is well demonstrated before in theory and experiments that for small-sized crystals like quantum wires, nanowires, etc., the quantum confinement effect will be very obvious and result in the downward frequency shift and linewidth broadening of the TO and LO phonon modes. Such change of phonon mode frequency and linewidth is mainly due to the relaxation of the *q* = 0 selection rule in the Raman scattering [14,15,22,29-33].

For better understanding of phonon properties in single NWs, excitation polarization-dependent Raman measurements were also performed on the single NWs. Figure 4c shows the Raman spectra of single NWs measured under four main polarization configurations ($x\left(z,z\right)\bar{x}$, $x\left(y,y\right)\bar{x}$, $x\left(z,y\right)\bar{x}$, and $x\left(y,z\right)\bar{x}$). It is observed that the intensity of the TO mode measured with parallel configuration, i.e., $x\left(z,z\right)\bar{x}$ and $x\left(y,y\right)\bar{x}$, when the incident and scattered light polarizations are parallel to each other, is much stronger than that with perpendicular configuration, and the intensity measured under the $x\left(z,z\right)\bar{x}$ configuration is much stronger than that under the $x\left(y,y\right)\bar{x}$ configuration. This indicates that the highest scattering intensity occurs when both the incident and analyzed light linear polarization are parallel to the NW growth axis. These results observed here are in accordance with those of ZB GaAs NWs reported in [16], which is mainly caused by the selection rules of the crystal. The excitation polarization-dependent Raman scattering measurements were performed by rotating the half-wave plate in 10° ± 2° increments and thus changing the angle, *ϕ*, between the electric vector of the incident light and the long axis of the NW. Figure 4d shows the polar scan of the intensity of the TO phonon mode of single InAs NWs as a function of the angle measured under two scattering configurations $x\left(\phi ,z\right)\bar{x}$ and $x\left(\phi ,y\right)\bar{x}$, where $x\|\left[1\bar{1}0\right],y\|\left[11\bar{2}\right],z\|\left[111\right]$. As shown in Figure 4d, for the $x\left(\phi ,z\right)\bar{x}$ configuration, the maximum intensity occurs at 5° and 175°, while the minimum intensity occurs at 85° and 265°. Some experimental points slightly deviate from the trend, which might be caused by the experimental artifact. For the $x\left(\phi ,y\right)\bar{x}$ configuration, there is a weakly preferential value of *ϕ* giving a maximum scattering intensity (maximum intensity is around 75° and minimum intensity is around 340°). It is noted that the maximum intensity measured under the $x\left(\phi ,z\right)\bar{x}$ polarization is around seven times that measured under the $x\left(\phi ,y\right)\bar{x}$ polarization, which indicates that the Raman scattering under the $x\left(\phi ,z\right)\bar{x}$ configuration is much more efficient than that under the $x\left(\phi ,y\right)\bar{x}$ configuration. This particular distribution of the maximum/minimum Raman peak intensity in the polar scan, as shown in Figure 4d, agrees well with that obtained with theoretical calculation for ZB InAs nanowires [23]. This further confirms that the InAs NWs studied here is mainly composed of ZB phase, which accords with the HRTEM results discussed before [16,23]. The TO mode of InAs NWs is found to act like a nearly perfect dipole antenna. The same behavior has been found in the other one-dimensional systems, such as SWNTs [34], 20-nm WS_2_ nanotubes [35], GaP NWs [26], and GaAs NWs [16]. The origin of this effect has been attributed to the scattering of the electromagnetic field from a dielectric cylinder of nanoscale dimensions [19]. Furthermore, it is observed that the light is preferentially absorbed when the incident light is polarized along the nanowire axis [36]. These theories about Raman selection rules and the one-dimensional geometry of the NW may be used to explain our experimental data.

## Conclusions

Raman scattering experiments have been performed on single InAs NWs. In the single NW spectra, a striking TO mode is observed at 215.8 cm^−1^, slightly lower than that of the reference bulk InAs (110) sample. This downward shift of the phonon frequency is mainly caused by defects or disorders that existed in the NW. The excitation polarization-dependent Raman measurements indicate that the TO phonon mode in the NW presents the highest scattering efficiency when both the incident and analyzed polarization are parallel to the NW growth axis. The TO mode of InAs NWs is found to act like a nearly perfect dipole antenna. This is a combined consequence of both the selection rules and the one-dimensional geometry of the NW.

## Abbreviations

HRTEM: High-resolution transmission electron microscopy; LO: Longitudinal optical; MOCVD: Metalorganic chemical vapor deposition; NWs: Nanowires; SEM: Scanning electron microscopy; TO: Transverse optical; ZB: Zinc blende.

## Competing interests

The authors declare that they have no competing interests.

## Authors’ contributions

TFL carried out the experimental analysis and drafted the manuscript. WL and LZG participated in the experimental analysis. LJG participated in its design and coordination. YHC carried out the experimental design. TY and ZGW participated in the experimental design. All authors read and approved the final manuscript.

## References

[B1] Yan RX Gargas D Yang PD Nanowire photonics Nature Photonics 2009 3 569 10.1038/nphoton.2009.184

[B2] Lu W Lieber CM Semiconductor nanowires J Phys D 2006 39 R387 10.1088/0022-3727/39/21/R01

[B3] Patolsky F Lieber CM Nanowire nanosensors Mater Today 2005 8 20

[B4] Li Y Qian F Xiang J Lieber CM Battery betters performance energy generation Mater Today 2006 9 18

[B5] Wei W Bao XY Soci C Ding Y Wang ZL Wang DL Direct heteroepitaxy of vertical InAs nanowires on Si substrates for broad band photovoltaics and photodetection Nano Lett 2009 9 2926 10.1021/nl901270n 19624100

[B6] Adachi S Properties of Group-IV, III-V and II-VI Semiconductors 2005 New York: Wiley

[B7] Dayeh SA Aplin D Zhou XT Yu PKL Yu ET Wang DL High electron mobility InAs nanowire field-effect transistors Small 2007 3 326 10.1002/smll.200600379 17199246

[B8] Jiang XC Xiong QH Nam SW Qian F Li Y Lieber CM InAs/InP radial nanowire heterostructures as high electron mobility devices Nano Lett 2007 7 3214 10.1021/nl072024a 17867718

[B9] Dick KA Caroff P Bolinsson J Messing ME Johansson J Deppert K Wallenberg LR Samuelson L Control of III-V nanowire crystal structure by growth parameter tuning Semicond Sci Technol 2010 25 024009 10.1088/0268-1242/25/2/024009

[B10] Hsu YF Xi YY Tam KH Djurisic AB Luo JM Ling CC Cheung CK Ng AMC Chan WK Deng X Beling CD Fung S Cheah KW Fong PWK Surya CC Undoped p-type ZnO nanorods synthesized by a hydrothermal method Adv Funct Mater 2008 18 1020 10.1002/adfm.200701083

[B11] Xiong QH Wang J Eklund PC Coherent twinning phenomena towards twinning superlattices in III-V semiconducting nanowires Nano Lett 2006 6 2736 10.1021/nl0616983 17163697

[B12] Algra RE Verheijen MA Borgstrom MT Feiner LF Immink G Enckevort WJP Vlieg E Bakkers EPAM Twinning superlattices in indium phosphide nanowires Nature 2008 456 369 10.1038/nature07570 19020617

[B13] Cardona M Guntherodt G Light Scattering in Solids II: Basic Concepts and Instrumentation 1982 Berlin: Springer

[B14] Adu KW Gutierrez HR Kim UJ Sumanasekera GU Eklund PC Confined phonons in Si nanowires Nano Lett 2005 5 409 10.1021/nl0486259 15755085

[B15] Adu KW Xiong Q Gutierrez HR Chen G Eklund PC Raman scattering as a probe of phonon confinement and surface optical modes in semiconducting nanowires Appl Phys A: Mater Sci Process 2006 85 287 10.1007/s00339-006-3716-8

[B16] Zardo I Conesa-Boj S Peiro F Morante JR Arbiol J Uccelli E Abstreiter G Morral AF Raman spectroscopy of wurtzite and zinc-blende GaAs nanowires: polarization dependence, selection rules, and strain effects Phys Rev B 2009 80 245324

[B17] Frechette J Carraro C Diameter-dependent modulation and polarization anisotropy in Raman scattering from individual nanowires Phys Rev B 2006 74 161404

[B18] Chen G Wu J Lu QJ Gutierrez HR Xiong QH Pellen ME Petko JS Werner DH Eklund PC Optical antenna effect in semiconducting nanowires Nano Lett 2008 8 1341 10.1021/nl080007v 18422362

[B19] Xiong Q Chen G Gutierrez HR Eklund PC Raman scattering studies of individual polar semiconducting nanowires: phonon splitting and antenna effects Appl Phys Mater Sci Process 2006 85 299 10.1007/s00339-006-3717-7

[B20] Livneh T Zhang J Cheng G Moskovits M Polarized Raman scattering from single GaN nanowires Phys Rev B 2006 74 03520

[B21] Li TF Chen YH Lei W Zhou XL Luo S Hu YZ Wang LJ Yang T Wang ZG Effect of growth temperature on the morphology and phonon properties of InAs nanowires on Si substrates Nanoscale Res Lett 2011 6 463 10.1186/1556-276X-6-463 21777417 PMC3211884

[B22] Begum N Bhatti AS Jabeen F Rubini S Martelli F Line shape analysis of Raman scattering from LO and SO phonons in III-V nanowires J Appl Phys 2009 106 114317 10.1063/1.3267488

[B23] Moller M Lima MM Cantarero A Dacal LCO Polarized and resonant Raman spectroscopy on single InAs nanowire Phys Rev B 2011 84 085318

[B24] Hormann NG Zardo I Hertenberger S Funk S Bolte S Doblinger M Koblmuller G Abstreiter G Effects of stacking variations on the lattice dynamics of InAs nanowires Phys Rev B 2011 84 155301

[B25] Yu PY Cardona M Fundamentals of Semiconductors 2005 Berlin: Springer

[B26] Wu J Zhang D Lu Q Gutierrez HR Eklund PC Polarized Raman scattering from single GaP nanowires Phys Rev B 2010 81 165415

[B27] Yazji S Zardo I Soini M Postorino P Morral AFI Abstreiter G Local modification of GaAs nanowires induced by laser heating Nanotechnology 2011 22 325701 10.1088/0957-4484/22/32/325701 21757796

[B28] Soini M Zardo I Uccelli E Funk S Koblmuller G Morral AFI Abstreiter G Thermal conductivity of GaAs nanowires studied by micro-Raman spectroscopy combined with laser heating Appl Phys Lett 2010 97 263107 10.1063/1.3532848

[B29] Gupta R Xiong Q Adu CK Kim UJ Eklund PC Laser-induced Fano resonance scattering in silicon nanowires Nano Lett 2003 3 627 10.1021/nl0341133

[B30] Piscanec S Cantoro M Ferrari AC Zapien JA Lifshitz Y Lee ST Hofmann S Robertson J Raman spectroscopy of silicon nanowires Phys Rev B 2003 68 241312

[B31] Adu KW Gutierrez HR Kim UJ Eklund PC Inhomogeneous laser heating and phonon confinement in silicon nanowires: a micro-Raman scattering study Phys Rev B 2006 73 155333

[B32] Lei W Chen YH Xu B Ye XL Zeng YP Wang ZG Raman study on self-assembled InAs/InAlAs/InP(001) quantum wires Nanotechnology 1974 2005 16

[B33] Campbell IH Fauchet PM The effect of microcrystal size and shape on the one phonon Raman spectra of crystalline semiconductors Solid State Commum 1986 58 739 10.1016/0038-1098(86)90513-2

[B34] Duesberg GS Loa I Burghhard M Syassen K Roth S Polarized Raman spectroscopy on isolated single-wall carbon nanotubes Phys Rev Lett 2000 85 5436 10.1103/PhysRevLett.85.5436 11136015

[B35] Rafailov PM Thomsen C Gartsman K Kaplan-Ashiri I Tenne R Orientation dependence of the polarizability of an individual WS2 nanotube by resonant Raman spectroscopy Phys Rev B 2005 72 205436

[B36] Wang JF Gudiksen MS Duan XF Cui Y Lieber CM Highly polarized photoluminescence and photodetection from single indium phosphide nanowires Science 2001 293 1455 1457 10.1126/science.1062340 11520977

